# Activation of the Tumor Suppressor PP2A Emerges as a Potential Therapeutic Strategy for Treating Prostate Cancer

**DOI:** 10.3390/md13063276

**Published:** 2015-05-27

**Authors:** Ion Cristóbal, Paula González-Alonso, Lina Daoud, Esther Solano, Blanca Torrejón, Rebeca Manso, Juan Madoz-Gúrpide, Federico Rojo, Jesús García-Foncillas

**Affiliations:** 1 Translational Oncology Division, Oncohealth Institute, IIS-Fundacion Jimenez Diaz, UAM, University Hospital “Fundacion Jimenez Diaz”, E-28040 Madrid, Spain; E-Mail: blanca.torrejonmoreno@gmail.com; 2 Group of Cancer Biomarkers, Pathology Department, IIS-Fundacion Jimenez Diaz, UAM, E-28040 Madrid, Spain; E-Mails: paula.galonso@fjd.es (P.G.-A.); daoudlina@gmail.com (L.D.); esthersbasket4@gmail.com (E.S.); rebeca.manso@fjd.es (R.M.); jmadoz@fjd.es (J.M.-G.)

**Keywords:** p-PP2A, CIP2A, okadaic acid, prostate cancer, therapy

## Abstract

Protein phosphatase 2A (PP2A) is a tumor suppressor complex that has recently been reported as a novel and highly relevant molecular target in prostate cancer (PCa). However, its potential therapeutic value remains to be fully clarified. We treated PC-3 and LNCaP cell lines with the PP2A activators forskolin and FTY720 alone or combined with the PP2A inhibitor okadaic acid. We examined PP2A activity, cell growth, prostasphere formation, levels of PP2A phosphorylation, CIP2A and SET expression, and AKT and ERK activation. Interestingly, both forskolin and FTY720 dephosphorylated and activated PP2A, impairing proliferation and prostasphere formation and inducing changes in AKT and ERK phosphorylation. Moreover, FTY720 led to reduced CIP2A levels. Treatment with okadaic acid impaired PP2A activation thus demonstrating the antitumoral PP2A-dependent mechanism of action of both forskolin and FTY720. Levels of PP2A phosphorylation together with SET and CIP2A protein expression were studied in 24 PCa patients and both were associated with high Gleason scores and presence of metastatic disease. Altogether, our results suggest that PP2A inhibition could be involved in PCa progression, and the use of PP2A-activating drugs might represent a novel alternative therapeutic strategy for treating PCa patients.

## 1. Introduction

Protein phosphatase 2A (PP2A) is a well-established tumor suppressor complex that plays a crucial role in regulating signaling pathways that are highly relevant in human cancer. In fact, several mechanisms of PP2A inhibition in cancer cells have been described, including alterations affecting all of the PP2A subunits and deregulation of endogenous PP2A inhibitors [1,2,3,4]. Interestingly, some relevant findings have recently been reported about the significance of PP2A inactivation in prostate cancer (PCa) [5,6,7,8]. It has been observed that PP2A downregulation is involved in castration resistance and induction of an aggressive phenotype in PCa cells, and that its restoration shows potent antitumor effects in both *in vitro* and *in vivo* models [5]. Overexpression of the PPP2CA gene led to a reduced migration and invasive potential of PCa cells, suggesting that PPP2CA suppresses aggressive PCa cell behavior [5]. These observations were confirmed with *in vivo* studies revealing that PPP2CA inhibits PCa cell growth and metastasis [5]. These results are in concordance with previous results by the same group showing that modulation of PP2A activity could represent a novel therapeutic approach in prostate cancer [6]. Furthermore, the existence of alterations affecting PP2A scaffold and regulatory subunits in this disease has been described [7,8]. Moreover, the endogenous protein Cancer Inhibitor of PP2A (CIP2A) has been reported to be highly expressed and involved in PCa progression via c-MYC regulation [9,10], and CIP2A knockdown is able to resensitize metastatic castration-resistant PCa cells to cabazitaxel [11]. However, contradictory results about the potential therapeutic value of PP2A activation in PCa have been reported to date [12,13,14,15,16]. Whereas some studies support the antitumor properties derived from PP2A activation of compounds such as sodium selenate, ceramide, or carnosic acid [12,13,14], others highlight that PP2A inhibition led to anticancer effects [15,16].

Despite the existence of data suggesting the relevance of PP2A activation status and its tumor-suppressor role in PCa, its potential therapeutic value as a molecular target in this disease requires clarification. Thus, it would be worthwhile to evaluate the antitumor effects of PP2A-activating drugs, which have shown their efficacy in other cancers with similar PP2A alterations, and their potential clinical use in PCa patients. In this work, we show that the PP2A activators forskolin and FTY720 (its unphosphorylated form) induced antitumor effects dependent on PP2A activation in PCa cells. The use of these drugs decreased cell growth; led to changes in PP2A, AKT, and ERK phosphorylation status and expression levels of the PP2A inhibitor CIP2A; and reduced prostasphere formation capability. Thus, these observations support the potential benefits that could be derived from the use of PP2A activators as an alternative therapeutic strategy in PCa.

## 2. Results

### 2.1. Forskolin and FTY720 Lead to Reduced Cell Viability in PCa Cells That Is Dependent on PP2A Activation

**Figure 1:**
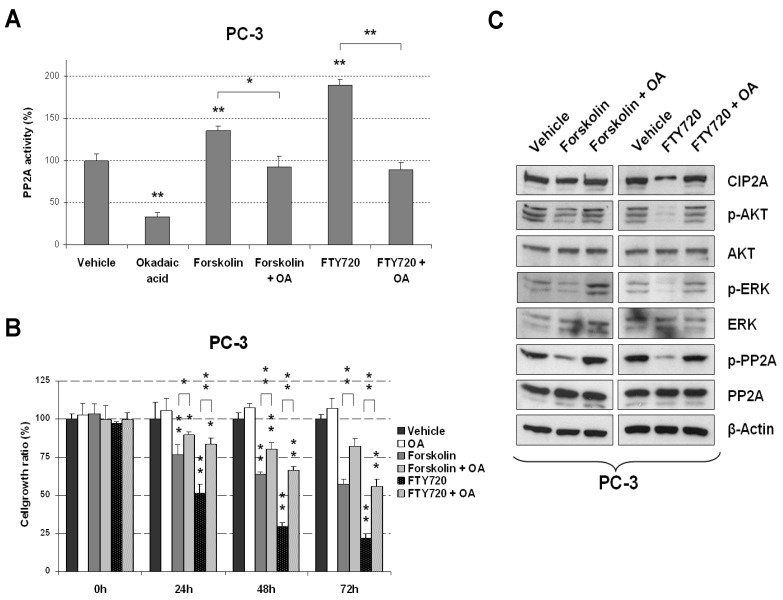
Forskolin and FTY720 impair PCa cell proliferation via PP2A activation. (**A**) PP2A assays in PC-3 cells treated with forskolin or FTY720, and pretreated or not with OA for 2 h; (**B**) MTS assay showing cell viability in PC-3 cells treated with forskolin or FTY720, alone or in combination with OA; (**C**) Western blot analysis of PP2A catalytic subunit (PP2Ac), CIP2A, SET, AKT and ERK1/2 after treatment with forskolin or FTY720 for 24 h in PC-3 cells; * *P* < 0.05; ** *P* < 0.01.

To study the potential therapeutic value of PP2A activation in PCa, PC-3 and LNCaP cells were treated with the PP2A activators forskolin and FTY720 or vehicle (DMSO). Phosphatase assays to quantify PP2A activity levels confirmed that forskolin and FTY720 treatment led to PP2A activation (Figure 1A and Supplementary Figure S1A). As a control, PCa cells were pretreated with the PP2A inhibitor okadaic acid (OA) for 2 h, followed by incubation with vehicle (DMSO), FTY720, or forskolin for 24 h. We observed that forskolin/FTY720-induced PP2A activity was inhibited by OA (Figure 1A and Supplementary Figure S1A). We next analyzed the effect of these PP2A-activating drugs on cell growth, observing a decreased proliferation in forskolin- or FTY720-treated PC-3 cells compared to vehicle-treated cells (Figure 1B and Supplementary Figure S2). Similar results were obtained using LNCaP cells (Supplementary Figures S1B and S3). In addition, we observed that the antiproliferative effects of forskolin and FTY720 were partially rescued by pretreatment with OA. Unexpectedly, we found that OA alone did not induce any significant effect on cell growth. However, similar observations have been reported in cell lines from several human tumors such as acute myeloid leukemia, colorectal or breast cancers, in which PP2A has been described to be inhibited [17,18,19]. One potential explanation for the lack of effect by OA alone could be a very high inactivated basal status of PP2A in the cell lines studied from these tumors. Altogether, these results show that PP2A activation by forskolin or FTY720 decreases proliferation in PCa cells.

### 2.2. Molecular Effects of PP2A Activation in PCa Cells after Forskolin or FTY720 Treatments

We next used western blot to analyze whether forskolin or FTY720 treatments could affect the phosphorylation status of previously described PP2A targets. We observed that both drugs induced decreased phosphorylation (activity) of AKT and ERK without affecting their expression levels. Moreover, OA treatment rescued AKT and ERK1/2 phosphorylation in forskolin- and FTY720-treated PC-3 cells (Figure 1C). Of interest, these effects on AKT and ERK were more evident in FTY720-treated cells. Similar results were observed in LNCaP cells (Supplementary Figure S1C).

We next analyzed PP2A, SET and CIP2A after treatment with forskolin or FTY720 alone or in combination with OA in PC-3 and LNCaP cell lines. Whereas the PP2A phosphorylation at Y307 was negatively affected by both forskolin and FTY720 treatments, only FTY720 was able to decrease CIP2A levels. No changes were observed in SET and PP2A expression (Figure 1C and Supplementary Figure S1C). In addition, the treatment with OA alone led to a slight increase of CIP2A protein (Supplementary Figure S4A). To further investigate the molecular mechanism by which FTY720 and OA modulate CIP2A, we quantified CIP2A mRNA by real-time PCR in PC-3 and LNCaP cells after treatment with FTY720 or OA. Interestingly, we found similar CIP2A levels in all cases, which suggests a translational regulation of CIP2A by these drugs (Supplementary Figure S4B).

### 2.3. Forskolin- and FTY720-Induced PP2A Activation Impair Prostasphere Formation

**Figure 2:**
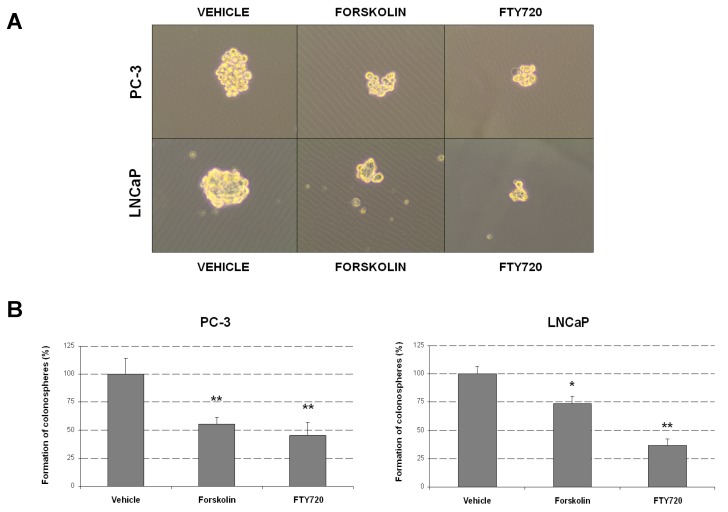
Forskolin/FTY720-induced PP2A activation reduces prostasphere-formation capability in PCa cells. (**A**) Optical microscope images (magnification 400×) showing representative prostaspheres derived from PC-3 and LNCaP cells after forskolin or FTY720 treatment; (**B**) FTY720 (5 µM) totally inhibits prostasphere formation in PC-3 and LNCaP cells; (**C**) Evaluation of the prostasphere formation after forskolin or FTY720 treatments alone or combined with OA in PC-3 and LNCaP cells; * *P* < 0.05; ** *P* < 0.01.

To further investigate the potential therapeutic effects of PP2A activation in PCa, we assessed prostasphere formation in PC-3 and LNCaP cells treated with forskolin or FTY720. We observed that both drugs led to decreased prostasphere-formation capability in PC-3 and LNCaP cells (Figure 2A). Interestingly, for this experiment, the concentration of FTY720 had to be reduced from 10 µM to 2.5 µM, since at higher concentrations prostasphere formation was totally inhibited (Figure 2B). In concordance with cell viability assays, pretreatment with OA partially restored the forskolin- and FTY720-induced antitumor effects (Figure 2C).

### 2.4. Deregulation of CIP2A and p-PP2A Are Associated with Aggressive Prostate Tumors

**Figure 3:**
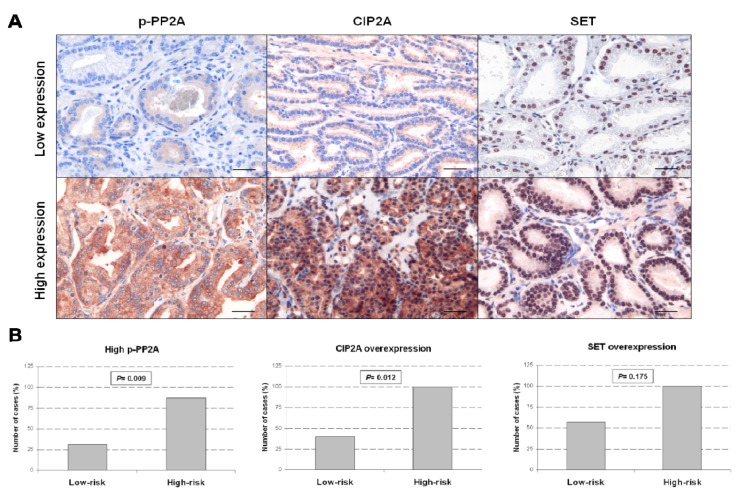
PP2A inhibition in patients with prostate cancer. (**A**) Immunohistochemical detection of p-PP2A, CIP2A and SET in PCa patients. The line in A and B shows 25 µm. Magnification 400×; (**B**) Association of CIP2A, p-PP2A and SET with low and high Gleason score tumors in 24 prostate cancer patients.

In order to assess whether the use of PP2A-activating drugs could be proposed as an alternative therapeutic strategy for treating PCa, we next evaluated CIP2A and p-PP2A in a set of 24 PCa patients. Patient characteristics are included in Supplementary Table S1 and immunohistochemical detection of CIP2A and p-PP2A is shown in Supplementary Figure S4. We found high CIP2A, p-PP2A and SET in 57%, 50% and 70% of cases, respectively. Patients were stratified in low- (6 or less) and high-risk (7–10) subgroups according to their Gleason score. Both CIP2A and p-PP2A levels were significantly elevated in the high-risk subgroup (Figure 3), and in those cases with metastatic disease (Supplementary Table S2). Altogether, these results suggest a potential involvement of PP2A inhibition in PCa progression.

## 3. Discussion

Although restoration of PP2A activity has emerged as an alternative therapeutic strategy for human leukemias in recent years [17,20], its potential therapeutic value in solid tumors remains comparatively underexplored. In fact, as various studies have highlighted the PP2A tumor-suppressor role in PCa, investigators have come to acknowledge that its molecular and clinical significance in this disease remains to be fully clarified. Interestingly, our observations, together with other novel findings in prostate cancer [5,6,7,8,9,10,11,12,13,14] and other recent observations in colorectal [18], pancreatic [21], and breast cancers [22], indicate that PP2A inhibition is a common alteration found in different solid tumors and further support the potential therapeutic benefits that would be derived from the inclusion of PP2A-activating drugs in anticancer protocols. Of relevance, it has recently been reported that PP2A is frequently inactivated in breast cancer patients. In concordance with our findings in prostate cancer, CIP2A overexpression and PP2A (Y307) hyperphosphorylation were identified as two main contributing alterations with relevant clinical and therapeutic implications in breast cancer [19]. Therefore, it remains urgent and necessary to develop clinical trials to test the promising preclinical effects shown by the treatment with PP2A activators in these tumor models. As mentioned above, some contradictory data can be found in the literature about the therapeutic implications derived from the pharmacological modulation of the PP2A activation status, thus making it necessary to clarify this point [12,13,14,15,16]. Of importance, our observations confirm a promising therapeutic value derived from the use of PP2A-activating drugs in PCa cells. Although both forskolin and FTY720 dephosphorylated and activated PP2A, we observed that FTY720 showed higher antitumor effects than forskolin. This observation is in concordance with the fact that FTY720 also induced higher PP2A activation than forskolin. One potential explanation could be that only FTY720 was able to decrease CIP2A levels (Figure 1 and Supplementary Figure S1). Furthermore, FTY720 has been reported to be a sphingosine kinase 1 inhibitor that reduces the expression of androgen receptor in prostate cancer cells [23,24]. Therefore, it would be very interesting to analyze the potential involvement of p-PP2A and CIP2A in this FTY720-induced effect.

As p-PP2A and CIP2A were modulated by the PP2A-activating drugs, we next analyzed their status in a set of PCa specimens. CIP2A is an endogenous PP2A inhibitor, and previous reports indicate that CIP2A overexpression is associated with high-risk Gleason PCa [9]. Interestingly, this observation was confirmed in our series, and we also identified a similar role for PP2A hyperphosphorylation (Figure 3). These results would indicate that PP2A inhibition status could be the critical alteration in determining PCa progression and may serve to define a subgroup of PCa patients with worse outcome. Those patients would be candidates to be treated with PP2A-activating drugs.

In conclusion, our results suggest that PP2A is a relevant molecular target for the development of novel therapeutic strategies based on the use of PP2A activators in PCa. Moreover, PP2A hyperphosphorylation and CIP2A overexpression are molecular mechanisms that contribute to inhibit PP2A in PCa and determine a more aggressive disease. However, the small number of cases included is a limitation and impairs assessment of the potential prognostic value that these alterations could have in PCa; therefore, their usefulness for prognosis and their ability to predict response to therapy in PCa patients should be further investigated in future studies with a larger cohort of cases.

## 4. Materials and Methods

### 4.1. Cell Cultures

The human PCa cell lines PC-3 (ATCC CRL-1435) and LNCaP (ATCC CRL-1740) were kindly provided by David Olmos (CNIO, Spain). Cell lines were maintained in RPMI-1640 (Invitrogen, Carlsbad, CA, USA) with 10% fetal bovine serum (FBS) and were grown at 37 °C in a 5% CO_2_ atmosphere. Media were supplemented with penicillin G (100 U/mL), and streptomycin (0.1 mg/mL). Cells were treated with the following reagents: FTY720 (10 µM), forskolin (40 µM), and OA (2.5 nM) (Calbiochem, San Diego, CA, USA).

### 4.2. Patient Samples

The study comprised consecutive formalin-fixed, paraffin-embedded samples of prostate tumor samples obtained from 24 patients who underwent radical prostatectomy. The samples were obtained from Fundacion Jimenez Diaz Biobank (BFJD, Madrid, Spain). Clinical data were collected by oncologists using patients’ clinical records. All investigations were carried out in accordance with the Declaration of Helsinki of 1975. Informed consent was provided in all cases. The ethical committee and institutional review board approved the project.

### 4.3. Western Blot Analysis

Protein extracts were isolated using TRIzol Reagent (Invitrogen, Carlsbad, CA, USA) following the manufacturer’s indications; these were then clarified (12,000× *g*, 15 min, 4 °C), denatured, and subjected to SDS-PAGE and western blotting. The antibodies used were mouse monoclonal anti-PP2A (Upstate Inc., Lake Placid, NY, USA), rabbit monoclonal anti-p-PP2A-C Y307 (Epitomics, Burlingame, CA, USA), rabbit polyclonal anti-AKT, rabbit polyclonal anti-ERK (Cell Signaling Technology Inc., Beverly, MA, USA), rabbit polyclonal anti-SET (Abcam, Cambridge, MA, USA), rabbit polyclonal anti-pAKT^Thr308^, rabbit polyclonal anti-pERK1/2^Thr202/Tyr204^ (Santa Cruz Biotechnology, Santa Cruz, CA, USA), rabbit polyclonal anti-CIP2A, and mouse monoclonal anti-β actin (Sigma, St. Louis, MO, USA). Proteins were detected with the appropriate secondary antibodies conjugated to alkaline phospatase (Sigma, St. Louis, MO, USA) by chemiluminescence using Tropix CSPD and Tropix Nitro Block II (Applied Biosystems, Foster City, CA, USA).

### 4.4. Proliferation Assay and Cell Viability

Cell proliferation was measured in triplicate wells by MTS assay in 96-well plates using the CellTiter 96 AQueous One Solution Cell Proliferation Assay (Promega, Madison, WI, USA) following the manufacturer’s indications.

### 4.5. PP2A Phosphatase Activity Assays

PP2A assays were performed with cell lysates (50 μg) using a PP2A immunoprecipitation phosphatase assay kit (Millipore, Temecula, CA, USA), following the manufacturer’s instructions.

### 4.6. Prostaspheres

For the generation of prostaspheres, 10,000 cells were plated in 6-well ultra-low attachment plates (Corning, New York, NY, USA). PC-3 and LNCaP cells were grown in serum-free medium DMEM/F12 + GlutMAX™-I (Gibco, Darmstadt, Germany) containing 1% N2 (Gibco, Darmstadt, Germany), 2% B27 (Gibco Darmstadt, Germany), 20 ng/mL human FGF (Sigma, St. Louis, MO, USA) and 50 ng/mL EGF (Sigma, St. Louis, MO, USA). After 7 days, the number of colonies formed was quantified to evaluate prostasphere formation.

### 4.7. Quantitative Real-Time RT-PCR

Total RNA was isolated using RecoverAll Total Nucleic Acid Isolation kit (Ambion, Austin, TX, USA) according to manufacturer’s instructions. Samples were reverse transcribed using the High Capacity Reverse Transcription Kit (Applied Biosystems, Foster City, CA, USA) and gene expression levels were quantified by quantitative real-time PCR using TaqMan Gene Expression Assays (Applied Biosystems, Foster City, CA, USA) specific for CIP2A and GAPDH as internal control. Relative gene expression was calculated according to the comparative cycle threshold (Ct) method.

### 4.8. Immunohistochemistry

Tissue sections (3 µm) were placed on plus-charged glass slides. After deparaffinization in xylene and graded alcohols, heat antigen retrieval was performed in pH 9 EDTA-based buffer (Dako, Hamburg, Germany). Endogenous peroxidase was blocked by 0.03% hydrogen peroxide for 5 min. Slides were incubated with the same primary antibody against CIP2A or p-PP2A for 60 min at room temperature, followed by the appropriate anti-Ig horseradish peroxidase-conjugated polymer (Flex+, Dako, Hamburg, Germany). Sections were visualized with 3,3′-diaminobenzidine as a chromogen. All stainings were performed in a Dako Autostainer. Sections incubated with normal non-immunized rabbit immunoglobulins were used as negative controls. CIP2A (1:5000), SET (1:5000) and p-PP2A (1:2000) antibody sensitivities had been calculated in a range of crescent dilutions of primary antibody. A semiquantitative histoscore was calculated by estimating the percentage of tumor cells positively stained with low, medium, or high staining intensity. The final score was determined after applying a weighting factor to each estimate. The following formula was used: histoscore = (low %) × 1 + (medium %) × 2 + (high %) × 3 and the results ranged from 0 to 300.

### 4.9. Statistical Analysis

Statistical analyses were performed using SPSS 20 for Windows (SPSS Inc., Chicago, IL, USA). Comparisons were carried out by chi-square and 2-sided *t*-test analyses. Data represented are mean of three independent experiments ± s.d. A *P* value less than 0.05 was considered statistically significant.

## Supplementary Files

Supplementary File 1
