# Supplementary material for: Activation of the Tumor Suppressor PP2A Emerges as a Potential Therapeutic Strategy for Treating Prostate Cancer
Source: Mar Drugs. 2015 May 27;13(6):3276–86. doi: 10.3390/md13063276 (PMC4483628; doi:10.3390/md13063276)
Supplement: Supplementary File 1 [file marinedrugs-13-03276-s001.pdf]

Supplementary Information

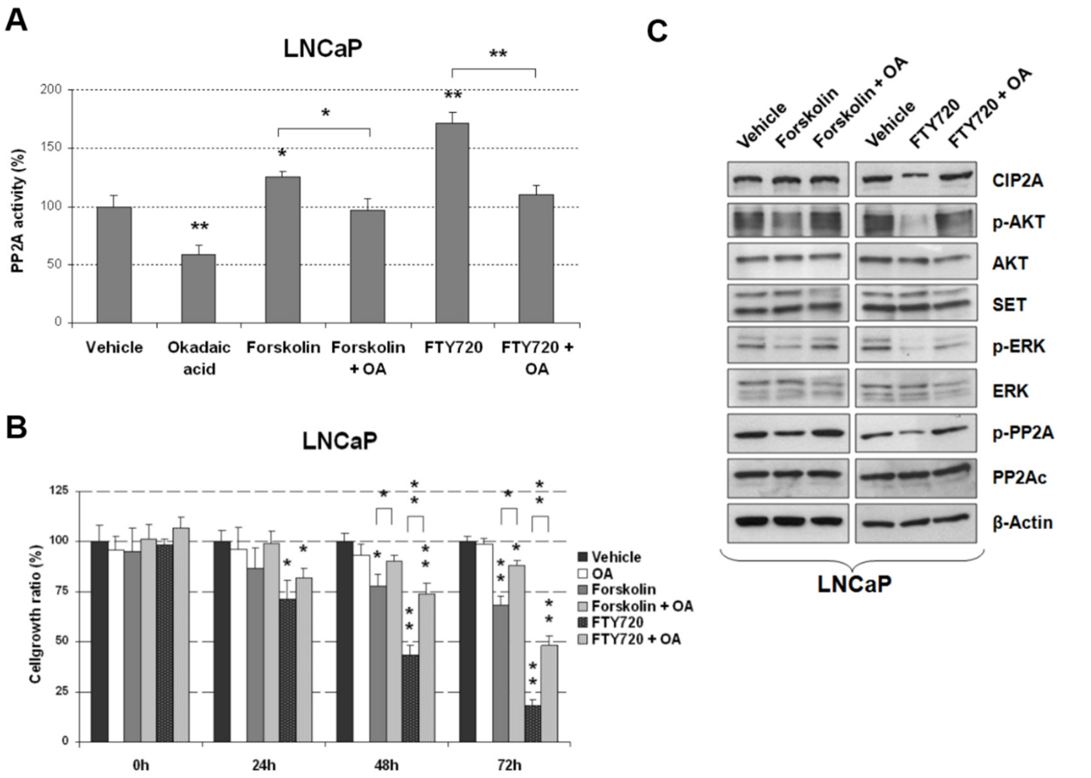

Figure S1. Forskolin and FTY720 impair PCa-cell proliferation via PP2A activation. (**A**) PP2A assays in LNCaP cells treated with forskolin or FTY720, and pretreated or not with OA for 2 h; (**B**) MTS assay showing cell viability in LNCaP cells treated with forskolin or FTY720, alone or in combination with OA; (**C**) Western blot analysis of PP2Ac, CIP2A, SET, AKT, and ERK1/2 after treatment with forskolin or FTY720 for 24 h in LNCaP cells; * $P < 0.05$; ** $P < 0.01$.

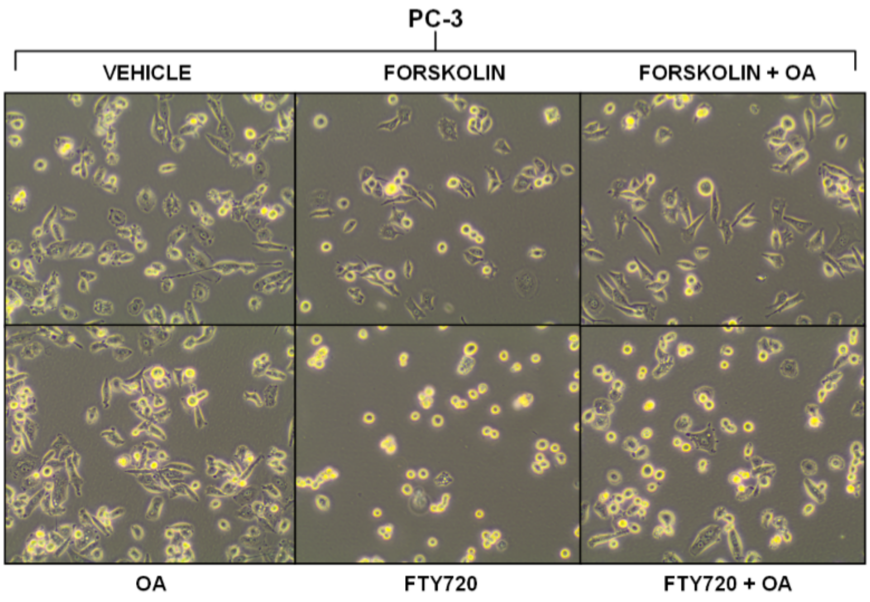

Figure S2. Optical microscope images showing PC-3 cells treated with forskolin or FTY720 for 72 h (magnification 400×).

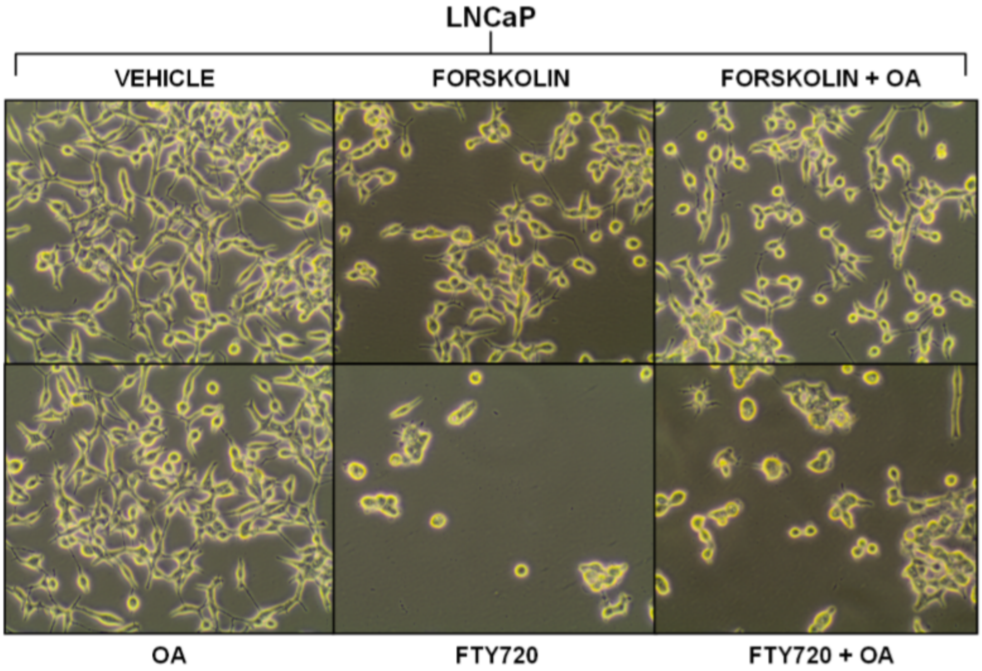

Figure S3. Optical microscope images showing LNCaP cells treated with forskolin or FTY720 for 72 h (magnification 400×).

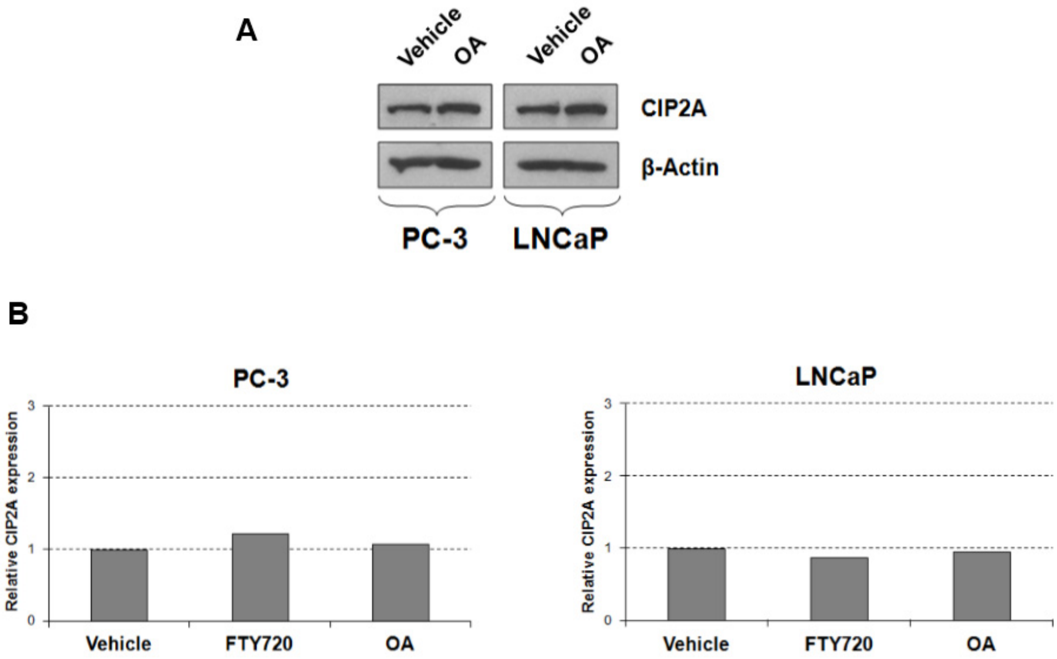

Figure S4. Regulation of CIP2A by FTY720 and OA. (**A**) Western blot analysis of CIP2A after OA treatment for 24 h; (**B**) Quantification of CIP2A mRNA by real-time PCR in PC-3 and LNCaP cells treated with FTY720 or OA.

Table S1. Clinical and molecular characteristics of the 24 patients with prostate cancer included in the study.

Case	Age	Gleasson	Metastases	Metastatic Site	RT	CT	HT	p-PP2A+	CIP2A+	SET+
P1	59	6 (3+3)	No		No	No	No	No	Yes	Yes
P2	69	6 (3+3)	No		No	No	No	No	No	ND
P3	69	6 (3+3)	No		No	No	No	No	No	Yes
P4	72	7 (3+4)	No		Yes	No	No	Yes	ND	ND
P5	67	6 (3+3)	No		No	No	No	Yes	No	ND
P6	65	6 (3+3)	No		No	No	No	Yes	No	ND
P7	60	6 (3+3)	No		No	No	No	No	No	Yes
P8	60	6 (3+3)	No		Yes	No	No	Yes	Yes	Yes
P9	57	6 (3+3)	No		No	No	No	Yes	Yes	No
P10	70	6 (3+3)	No		No	No	No	Yes	Yes	No
P11	69	6 (3+3)	No		Yes	No	No	No	No	No
P12	71	6 (3+3)	No		No	No	No	No	No	ND
P13	64	6 (3+3)	No		No	No	No	No	Yes	ND
P14	65	6 (3+3)	No		No	No	No	No	No	ND
P15	65	6 (3+3)	No		No	No	No	No	Yes	ND
P16	68	7 (3+4)	No		No	No	No	No	Yes	ND
P17	73	6 (3+3)	No		No	No	No	No	No	ND
P18	63	6 (3+3)	No		No	No	No	No	ND	ND
P19	63	7 (3+4)	Yes	Multiple sites	No	No	Yes	Yes	Yes	Yes
P20	80	9 (4+5)	Yes	Lymph nodes	No	No	Yes	Yes	Yes	Yes
P21	78	7 (3+4)	Yes	Gall bladder	Yes	No	Yes	Yes	ND	ND
P22	71	8 (3+5)	Yes	Lymph nodes	Yes	No	Yes	Yes	Yes	ND
P23	68	8 (3+5)	Yes	Bone	No	Yes (docetaxel)	Yes	Yes	Yes	ND
P24	66	7 (3+4)	Yes	Bone	No	No	No	Yes	Yes	Yes

RT: radiotherapy; CT: chemotherapy; HT: hormone therapy; ND: no data.

Table S2. Association of CIP2A, p-PP2A and SET with the presence of metastatic disease in 24 prostate cancer patients.

	No. Cases	NMD (%)		MD (%)		*P*
CIP2A overexpression	21	16		5		0.027
No	9	9	(100)	0	(0)	
Yes	12	7	(58.3)	5	(41.7)	
PP2A hyperphosphorylation	24	18		6		0.005
No	12	12	(100)	0	(0)	
Yes	12	6	(50)	6	(50)	
SET overexpression	10	7		3		0.175
No	3	3	(100)	0	(0)	
Yes	7	4	(57.1)	3	(42.9)	

NMD: non-metastatic disease; MD: metastatic disease.

© 2015 by the authors; licensee MDPI, Basel, Switzerland. This article is an open access article distributed under the terms and conditions of the Creative Commons Attribution license (http://creativecommons.org/licenses/by/4.0/).
